# Relevance of animal models to human tardive dyskinesia

**DOI:** 10.1186/1744-9081-8-12

**Published:** 2012-03-09

**Authors:** Pierre J Blanchet, Marie-Thérèse Parent, Pierre H Rompré, Daniel Lévesque

**Affiliations:** 1 Faculty of Dental Medicine, University of Montreal, PO Box 6128, Succ. Centre-ville, Montreal, QC, H3C 3J7, Canada; 2 Central Nervous System Research Group, University of Montreal, PO Box 6128, Succ. Centre-ville, Montreal, QC, H3C 3J7, Canada; 3 University of Montreal Hospital Centre (C.H.U. Montreal), 1560 Sherbrooke St. East, Montreal, QC, H2L 4M1, Canada; 4 Louis-H. Lafontaine Hospital, 7401 Hochelaga St., Montreal, QC H1N 3M5, Canada; 5 Faculty of Pharmacy, University of Montreal, PO Box 6128, Succ. Centre-ville, Montreal, QC, H3C 3J7, Canada

**Keywords:** tardive dyskinesia, stereotypies, vacuous chewing movements, antipsychotic drugs, dopamine receptors, non-human primates

## Abstract

Tardive dyskinesia remains an elusive and significant clinical entity that can possibly be understood via experimentation with animal models. We conducted a literature review on tardive dyskinesia modeling. Subchronic antipsychotic drug exposure is a standard approach to model tardive dyskinesia in rodents. Vacuous chewing movements constitute the most common pattern of expression of purposeless oral movements and represent an impermanent response, with individual and strain susceptibility differences. Transgenic mice are also used to address the contribution of adaptive and maladaptive signals induced during antipsychotic drug exposure. An emphasis on non-human primate modeling is proposed, and past experimental observations reviewed in various monkey species. Rodent and primate models are complementary, but the non-human primate model appears more convincingly similar to the human condition and better suited to address therapeutic issues against tardive dyskinesia.

## Introduction

Tardive dyskinesia (TD) is a disabling and potentially irreversible motor complication encompassing all persistent, abnormal, involuntary hyperkinetic movements occurring in the setting of chronic therapy with dopamine receptor-blocking agents, such as antipsychotic drugs and metoclopramide [1]. The resulting movement disorder is most often stereotyped in nature and typically involves the orobuccolingual musculature. As TD remains an elusive drug complication over 50 years since its initial description, it is not surprising that the treatment options available are non-specific and produce mixed results.

Unfortunately, the early hope that second-generation (so-called atypical) antipsychotic drugs would afford a gradual disappearance of TD has been challenged by recent reports that one third of patients chronically exposed to antipsychotic drugs still develop TD [2-4]. The annual risk remains greater in older adults, particularly in those living with a dementing illness [5]. Severe forms of TD also develop with the new antipsychotic drugs [6]. The persistence of TD in the community, and the enlarging spectrum of conditions for which antipsychotic drugs are prescribed (e.g., bipolar disorder, refractory depression), make it urgent to develop a better understanding of this hyperkinetic movement disorder, as well as novel preventive and palliative approaches. The use of animal models is inescapable and irreplaceable in reaching that goal. The advances made since the levodopa-induced dyskinesia primate model was introduced in neuroscience 25 years ago well illustrate that point [7,8]. Before mechanistic considerations are addressed, any experimental movement disorder model must first reproduce the phenomenology of the human condition, a test the levodopa-induced dyskinesia primate model has unequivocally passed. The purposeless hyperkinetic movements should be reproducible, quantifiable, and easily distinguishable from normal movements. This personal review of rodent as well as non-human primate TD models provides an opportunity to reflect upon the diversity of protocols, and lack of consensus thereof, and to suggest the most fruitful approaches. Cats and guinea pigs have rarely been used. We also take advantage of this exercise to compare the existing literature with our own recent experience with non-human primate modeling.

### Rat models

The use of rats exposed to antipsychotic drugs for consecutive weeks has been proposed with variations since the early 70's. Originally, brief exposure of guinea pigs to chlorpromazine was followed by abrupt withdrawal and challenge with dopamine receptor agonists, which triggered stereotyped gnawing and sniffing [9]. The rapid onset of the motor stereotypies and need to sequentially use dopamine antagonist and agonist drugs certainly do not replicate the delayed course and spontaneous occurrence of TD in humans treated with dopamine receptor-blocking agents. Many laboratories have then characterized the spontaneous oral movements emerging during subchronic or chronic antipsychotic drug administration. The behavioural results obtained have been summarized in authoritative literature reviews [10,11], which documented different patterns and descriptors of oral movements, most commonly reported as robust, seemingly purposeless, chewing activity (so-called vacuous chewing movements or VCM), often fluctuating and occurring in short bursts, occasionally associated with bruxism and tongue protrusions. Rapid wide mouth openings and bursts of oromandibular tremor or twitching may be distinguished. In most reports, the proportion of animals displaying high VCM scores range from 30% to 50% according to the type of antipsychotic used, as well as the route of drug administration [11-18].

The VCMs and tongue protrusions are typically counted during repeated 2- to 5-min session blocks in an observation cage equipped with a mirror placed in the back to improve visibility, after a variable habituation period to the new environment (up to 1 h), in single freely moving animals without access to food. Other investigators prefer to use stopwatches to record burst duration rather than single events. An observation tube, restraining the animal placed in front of a camera focused on the head, has been tested to optimize motor assessment, but requires much longer habituation to reduce stress effects [19]. This can be combined with a computer-assisted detection system to measure oral movements that cannot be visually quantified, but does not obviate the need for visual examination to distinguish purposeless chewing from bursts of jaw tremor or behavioural stereotypies temporally related to grooming or gnawing. Although drug-induced hypoactivity of the animal in the cage may facilitate and bias VCM detection, no significant change in locomotion was documented between VCM and VCM-free rats in one study, and grooming behaviour was not considered a hindrance to VCM counting [20]. In contrast to human TD, extraoral dyskinesia has not been observed.

It is important but complex to distinguish early-onset from gradual late-onset VCMs in rodents during exposure to antipsychotic drugs. Early VCMs emerge rapidly (1-21 days), are sensitive to anticholinergic drugs, and may more closely reflect acute extrapyramidal symptoms (EPS) (parkinsonian tremor, dystonia), whereas more delayed VCMs (> 3 months) are insensitive to anticholinergics, and may better reflect tardive dyskinesia [10,12,21]. These two patterns are also associated with differences in neuropeptide gene expression in the striatum [21]. Thus, although similar from a behavioural perspective, early- and late-onset VCMs appear to have different pharmacological and neurochemical profiles. Unfortunately, the transitional time point between early- and late-onset VCMs remains elusive. It is suggested that rats with late-onset high-VCM drug responses should be included in TD studies. In contrast to brief drug exposure protocols, long-term exposure seems more likely to promote the persistence of the VCM response following drug withdrawal, typically vanishing over a 2-week follow-up period [22], although longer persistence for several months has been documented [10,23].

In contrast to levodopa-induced dyskinesia, continuous administration using depot formulations (e.g., haloperidol decanoate i.m.) produces a stronger VCM response compared to daily, dose-equivalent (i.p.), intermittent drug administration [11]. The importance of this observation is exemplified by the fact that continuous, but not intermittent, antipsychotic drug administration, delivered through a subcutaneous osmotic minipump, has generated high VCM scores, in spite of similar total daily doses [24,25]. Continuous levels of dopamine D_2 _receptor occupancy during antipsychotic drug exposure increase the risk of receptor upregulation [26,27], and could trigger distinct drug-induced neuroadaptation. Indeed, continuous, but not intermittent, haloperidol administration exacerbates psychostimulant drug reward behaviours and is associated with an aberrant psychostimulant-induced striatal gene regulation [28].

Since long-term drug exposure is not cost- and time-efficient, several laboratories still use a 3-week drug protocol to generate a VCM response, while others have attempted to find ways to accelerate and magnify rat TD-like oral activity with less variability. Old rats have been tested. Indeed, advancing age is the single most significant risk factor for early TD induction and persistence [29-31]. Old rats generally show a reduced level of spontaneous motor activity, but a higher level of oral movements in the unmedicated state, compared to young rats. During antipsychotic drug exposure, they may show a stable (ceiling effect) or heightened VCM response. It is unclear whether the use of aged rats provides a distinct advantage in experimental TD protocols [14,32,33]. Of practical importance in study design, certain strains such as the Long-Evans rats display a more robust VCM response in all treated individuals, whereas 38% of Sprague-Dawley rats never express VCM over 24 weeks [34]. Unlike in the human literature, the impact of gender and diabetes on experimental TD has been little addressed. One study reported a heightened VCM response in female mice that was inconsistent at different time points [35]. The influence of glucose metabolism on the VCM induction process and severity has not been investigated, but a surprising apparent reduction in apomorphine-enhanced VCM has been observed in diabetic rats exposed to haloperidol compared to control rats [36]. A few attempts to enhance the susceptibility to TD in rats with pre-existing central nervous system injury have been published [37,38]. While unnatural and remote from the experience of most people developing TD, these experiments may still provide clues about TD induction. Frontal cortex lesions generated more severe and persistent abnormal oral behaviours in adult rats after antipsychotic drug withdrawal [37], and neonatal dopamine denervation with 6-hydroxydopamine had an even greater impact on the severity of the VCM response triggered by haloperidol, which persisted for 8 months following drug withdrawal [38]. Partial dopamine loss may thus constitute a sensitization stage prior to the precipitation stage resulting from drug exposure, but whether this has some bearing on the vulnerability to TD seen in normal aging is undetermined. The sensitization stage may trigger additional pathogenic mechanisms contributing to the drug-induced neural changes conducive to TD, such as the sensitivity of dopamine D_1 _and serotonin 5-HT_2C _receptors [38], and/or lessening the endogenous adaptive response mounted to fend off TD.

One finding raising concern about the relevance of the VCM response is the apparent similar behaviour produced by the acute (3 days) or chronic (4-6 weeks) administration of the presynaptic monoamine depleter reserpine. This drug can trigger VCM, tongue protrusions, and facial twitching, which persist for periods extending up to 2 months following drug withdrawal [39]. These oral movements were more persistent in old than in young animals following drug withdrawal [32]. No general consensus exists concerning the validity of the reserpine model.

Thus, chronic antipsychotic drug exposure (typically with haloperidol) for at least 3-4 months appears to represent a standard approach to model TD in rats. The VCM response certainly does not display the complexity of human TD, and may appear very early in some animals during drug exposure. The model still provides insights about the human disorder, and has been proposed in experiments using antisense oligonucleotides to knockdown site-directed single target proteins that may be closely associated with the VCM response. For instance, intrastriatal infusion of oligonucleotide antisense to dopamine D_1A _receptor mRNA has selectively reduced receptor binding and VCMs in rats exposed to fluphenazine decanoate [40].

It is well established that haloperidol and other typical antipsychotic drugs (D_2 _receptor antagonists) upregulate dopamine D_2 _receptors. This led to the suggestion that late-onset dyskinesia was a reflection of dopamine receptor supersensitivity [9]. However, several observations indicate that D_2 _receptor upregulation alone is not sufficient to account for TD. In rats, haloperidol-induced D_2 _receptor upregulation develops early during treatment (within 15 days), while tardive VCMs only develop after much prolonged treatment [41,42]. Furthermore, D_2 _receptor upregulation similarly occurs in haloperidol-treated rats showing no or low VCMs and those displaying high VCM scores [11]. Similarly, no difference in striatal D_2 _receptor binding has been observed in patients with TD compared to antipsychotic drug-treated patients without TD [43].

Morphological changes observed in rats exposed to antipsychotics with high VCM scores included alterations in striatal synaptic morphology [15,18], medial substantia nigra pars compacta nerve cell loss and atrophy particularly in aged rats [14], and alterations in nucleus accumbens dendritic spine density [16], but decreased spine density was not associated with VCM in the striatum [17]. Reductions in striatal choline acetyltransferase activity [44,45] and in the number of large striatal (presumably cholinergic) interneurons [45,46] have been reported following chronic antipsychotic drug exposure in rats, but the relevance of the results to TD and distinction from age-related effects remain unclear. Neurochemical data seem to point out to an important role of striatal dynorphin expression and/or increased glutamatergic activity [13,15,16,18,21]. However, the latter observations are correlative in nature, and no study designed to evaluate a causal link between these changes and induction of high VCM scores has been conducted so far.

### Transgenic mouse models

Transgenic mice consist of strains whose genetic material has been deliberately modified using foreign DNA transfer into the host embryo by recombinant DNA technology. They are used to study the contribution of knockout genes on the response to antipsychotic drugs and on motor complications in particular. In the past, D_3 _receptor knockout mice have been tested to look at striatal gene and protein expression following the administration of different antipsychotic drugs [47,48]. We have examined *Nur77 *knockout mice and characterized their VCM response following the administration of haloperidol for up to 17 weeks [49]. *Nur77 *(NGFI-B, NR4A1) is a transcription factor of the nuclear receptor family expressed in dopaminoceptive circuits, whose striatal expression is upregulated by haloperidol in striatopallidal neurons [50,51]. Interestingly, *Nur77 *knockout mice display spontaneous VCMs in the unmedicated state and a stronger VCM response following haloperidol exposure compared to wild type mice (see accompanied Additional file 1: Table S1) [49]. This suggests that neurochemical changes induced by genetic deletion of *Nur77 *might have recapitulated some susceptibility processes observed in humans with TD. We also showed that a single nucleotide polymorphism (SNP, rs2603751) located in the 3'-UTR (untranslated regulatory region) of the *Nur77 *mRNA displayed a nominal association with the risk of developing TD, as well as with TD intensity based on Abnormal Involuntary Movement Scale (AIMS) scores in a group of schizophrenia patients [52]. More studies are underway to shed light on the signalling partners and cellular effects of this nuclear receptor in the basal ganglia, in normal and drug-primed conditions.

### Non-human primate model

**Figure 1:**
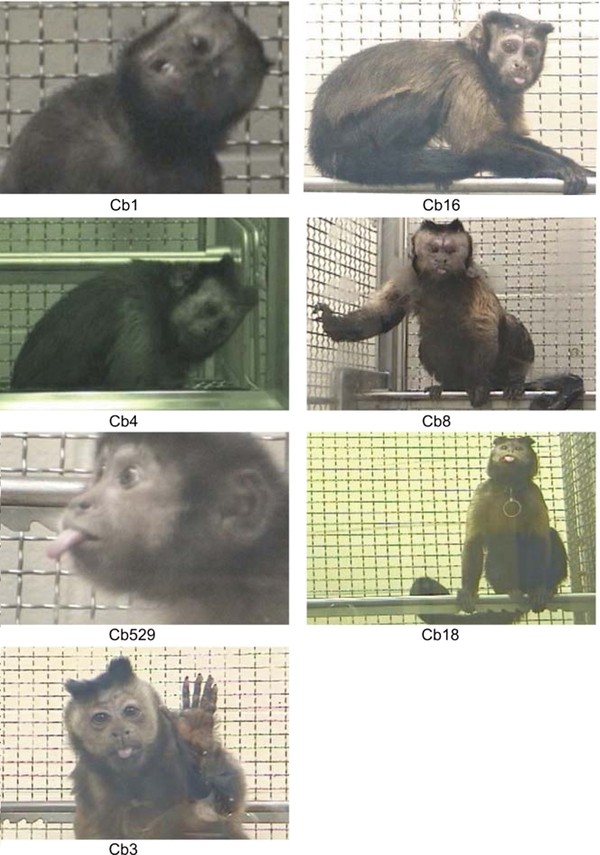
**Seven capuchins displaying tardive dyskinetic movements causing variable tongue protrusions, as well as forehead contractions, lip retraction, and neck twisting**.

The initial attempts to model TD in macaques were not encouraging, with impermanent dyskinetic signs often admixed with acute dystonic reactions [54,55]. For these reasons, this TD model has been underused compared to the levodopa-induced dyskinesia primate model, explaining in part the existing gap in knowledge between the two iatrogenic disorders. With careful antipsychotic drug exposure, the abnormal movements observed are similar to those found in humans and typically stereotyped in nature, without concomitant acute dystonic reaction or interference with food intake and well-being (see accompanied Additional file 1: Table S1). Variable orofacial dyskinetic movements are seen (6 of 7 TD capuchins in our laboratory), including forehead contractions, chewing movements, tongue protrusions, and lip retraction (Figure 1). Neck rotation, brief back extension, and flexion/extension movements of the toes, are also seen. Upper limb chorea was occasionally observed. In some reports, the abnormal movements have persisted for several months following drug withdrawal.

Although the review of 21 published studies suggests marked interspecies differences in susceptibility that should be interpreted with caution in view of the various drug protocols used (see Additional file 2), the TD rate in primates is favourable and exceeds the annual incidence of 3-5% generally reported in human adults [74]. A cumulative account of the original experiments that have used *Macaca *species indicates that only 8% (10/122) of the subjects chronically exposed to antipsychotic drugs alone developed TD. The mean (± SD) drug exposure of 14.1 (± 14.8) months was somewhat shorter than in capuchins. Baboons do not tolerate haloperidol well, and it is unknown whether they may safely be exposed to other first-generation antipsychotic drugs. Nonetheless, the dehydrated derivative of haloperidol, 4-(4-chlorophenyl)-1-[4-(4-fluorophenyl)-4-oxobutyl]-1,2,3,6-tetrahydropyridine (HPTP), was chronically administered with success to these animals and rapidly triggered TD [64]. In three New World monkey species, squirrels (*Saimiri sciureus*), capuchins (*Cebus apella*) and marmosets (*Callithrix jacchus*), TD developed in proportions of 0% (0/5 animals in a single study), 45% (32/71, including 7/18 observed in our laboratory) and 71% (12/17 in two studies), respectively. The onset of TD is unpredictable and highly variable in capuchins as well as in marmosets, with a mean latency of onset of 33.4 (± 22.9) months and 18.0 (± 8.5) months, respectively. In our 7 TD capuchins, the mean latency of onset was 17 months (median, 10 months; range 3-35 months). Interestingly, capuchins carry the gly9 polymorphism in the dopamine D_3 _receptor gene [75] that has been associated with TD in humans [76]. This genetic trait has not been sought in marmosets. The differential susceptibility of various non-human primate species to TD is reminiscent of the variable prevalence in TD reported in humans of diverse ethnic background, a very complex issue lacking conclusive evidence at present [77].

In the drug protocols examined (Additional file 2), haloperidol and fluphenazine were most often used and the duration of exposure generally extended to at least one year. Chronic oral drug administration was slightly more effective at triggering TD in capuchins than depot i.m. preparations, with incidence of 53% and 42%, respectively, but the cumulative drug exposure resulting from these two drug regimens may well have differed. On the other hand, in common marmosets exposed to haloperidol [72,73], a depot i.m. preparation more effectively triggered TD than oral administration, a result which could be in line with the rat data discussed previously. Although the interrupted or sustained nature of the drug protocols pursued did not appear to correlate with TD susceptibility, temporary drug suspension did trigger persistent withdrawal-emergent TD in some animals [78,61], including 3 of our 18 capuchin monkeys. Thus, it appears reasonable to suspend drug exposure from time to time, at least to ensure that TD is not masked by parkinsonian features.

**Figure 2:**
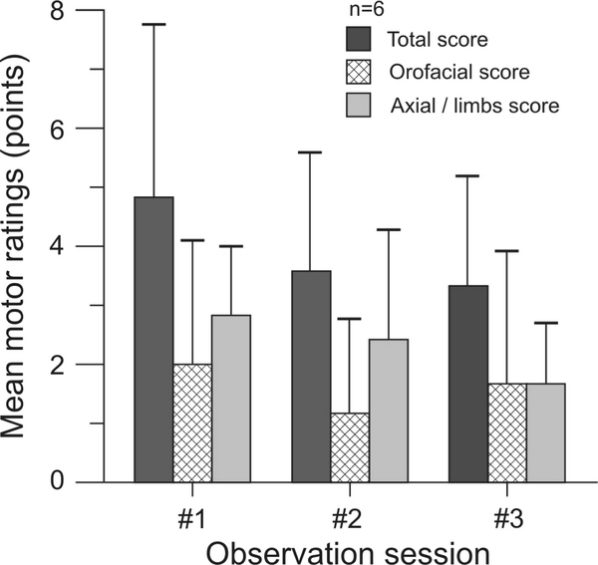
**Mean (± SD) tardive dyskinesia scores obtained in 6 animals rated on 3 different occasions 4 weeks apart**. Individual scores represent the sum of several body parts. The mean coefficient of variation calculated from this data set with normal distribution is 31.5%.

The recognition and rating of TD are easy in non-human primates [62,72]. We have used a primate equivalent of the Abnormal Involuntary Movements Scale (AIMS) to blindly score body segments (orofacial [forehead, lips, tongue, jaw], neck, trunk, and each limb) between 0-4 points (absent to severe intensity), for a maximum score of 40 points. The intensity of TD in our animals was generally mild, and orofacial scores accounted for approximately 40% of the total TD score. As reported previously [61], stress enhances TD and should be kept constant as much as possible. Reminiscent of the diurnal variability in orofacial TD measured in a fraction of TD patients [79], TD intensity showed some variability in the short term (Figure 2), and the spontaneous dispersion of TD ratings (N = 6) in 3 data sets of normal distribution generated 4 weeks apart revealed a mean coefficient of variation of 31.5% (range 10.2-49.5). We calculated that a sample size of 7 monkeys would be necessary to demonstrate the antidyskinetic properties of an investigational drug, taking into account the spontaneous variability in TD scores, and a power of 80% and alpha value of 0.05 to detect an antidyskinetic effect size of at least 50% relative to baseline scores.

Although the TD incidence in capuchin monkeys exceeds several-fold that in humans and macaques, the unpredictability, long delay of induction, and cost of this experimental TD model, constitute impediments to broad applications. As in the rodent model, certain factors have been proposed to accelerate experimental TD induction. High drug dosing may seem desirable to sensitize basal ganglia circuits and promote TD induction (Additional file 2), but this often triggers acute dystonic reactions even during maintenance of drug exposure [80]. Thus, we have elected to administer low i.m. weekly doses. Such dosing frequency could better account for the faster metabolism and clearance of haloperidol reported in non-human primates compared to humans [81]. It is unknown whether antipsychotic drug disposal in rhesus is faster than in capuchins, but this could in part explain their apparent low TD susceptibility. As in rats, acute drug challenge with a dopamine agonist has been proposed in monkeys as a way to reflect the behavioural sensitivity of animals chronically exposed to antipsychotic drugs and enhance experimental TD, but the impact of this strategy wanes over time and its validity as a model has been questioned [82]. The age of the animals has been little examined [61]. Since the availability of monkeys over 24 years of age is limited, this strategy is not widely applicable. Bilateral ovariectomy was first performed in our females to reduce gonadal hormone modulation on brain dopamine and GABA receptors during the estrous cycle [83-86], as proposed in the levodopa-induced dyskinesia primate model [87]. Although it is intuitively beneficial to reproduce the postmenopausal hormonal status of women most at risk for TD [88], this procedure does not make a senescent brain and its place as a TD-enhancing strategy remains undetermined. The use of common marmosets has also been suggested since they tend to display TD sooner (see Additional file 2), often within the first year after antipsychotic drug initiation. This carries definite advantages in lowering cohort size and cost, but the exceptionally high cumulative TD incidence in that species, reaching 100% in one report [72], raises some concerns about the immediate applicability of the marmoset model to human TD. Other strategies could eventually be developed to increase TD susceptibility in monkeys, with the aim to dampen neuroadaptive mechanisms [49], or inversely, to amplify abnormal molecular signalling triggered by antipsychotic drugs [89-92].

Neuropathological examination of baboons with TD following exposure to the dehydrated product of haloperidol revealed no obvious changes in neuronal density and neuropeptide content (substance P, enkephalin) in the basal ganglia relative to control animals, but gliosis in the caudate nucleus was observed in the TD group [64]. A 25% reduction in density of the magnocellular cholinergic neurons was found in the anterior portion of the nucleus basalis of Meynert in the TD brains only, while the striatal cholinergic interneurons were spared. The functional impact of this loss on the activity of the cortico-subcortical circuits, and on the subthalamic nucleus in particular, remains undetermined. The regional reductions in glutamate decarboxylase (GAD) activity found initially in the substantia nigra only in capuchins with severe TD left untreated for 2 months before sacrifice, affecting to a lesser extent the medial globus pallidus and subthalamic nucleus [80,70], were not consistently replicated in subsequent studies [91]. A single 2-deoxyglucose study involving 4 capuchins with chronic TD left untreated for 4 months showed decreased uptake levels in the medial globus pallidus and ventral (VA/VL) thalamus compared to 3 controls, suggesting parallel changes in synaptic activity within the inputs to these structures susceptible to promote dyskinesia [92].

In conclusion, the animal models of TD discussed are complementary and offer different ways to examine various aspects of antipsychotic drug responses. The latency of onset, individual susceptibility, phenomenologic expression of the purposeless movements, and persistence of TD signs between drug dosing, make the non-human primate TD model convincingly similar to the human condition, and best suited to address therapeutic issues. In our view, this model will continue to contribute to our understanding of TD.

### Financial Disclosure of all authors (for the preceding 12 months)

Pierre Blanchet and Daniel Lévesque have received research funding from the Canadian Institutes of Health Research (Ottawa) and the National Alliance for Research on Schizophrenia and Depression (NARSAD). P. Blanchet also received speaker fees from Biovail Pharmaceuticals Canada, Novartis Pharma Canada, and Teva Canada Innovation. Marie-Thérèse Parent and Pierre Rompré have no independent public or private source of financial support to declare.

## Competing interests

The authors declare that they have no competing interests.

## Authors' contributions

All authors read and approved the final manuscript.

## Supplementary Material

Additional file 1 **Table S1**. Experience of chronic antipsychotic drug exposure in non-human primates [53-56,58,62-65,80,83-92]. Click here for file

Additional file 2 **Segment #1 shows typical haloperidol-induced vacuous chewing movements in a wild type mouse**. Occasional, brief vertical jaw movements are observed. The next segments show six different capuchin monkeys still exposed to a maintenance intramuscular weekly dose of antipsychotic drug when the videoclips were captured. The phenomenology of the purposeless movements includes variable orofacial, axial, and foot dyskinesias, at times admixed with mild dystonic features. The animals are otherwise in good condition and able to feed, groom, ambulate, and interact with peers. Click here for file
