# Supplementary material for: Relevance of animal models to human tardive dyskinesia
Source: Behav Brain Funct. 2012 Mar 9;8:12. doi: 10.1186/1744-9081-8-12 (PMC3338072; doi:10.1186/1744-9081-8-12)
Supplement: Additional file 1 — Table S1. Experience of chronic antipsychotic drug exposure in non-human primates [53-56,58,62-65,80,83-92]. [file 1744-9081-8-12-S1.DOC]

Table. Experience of chronic antipsychotic drug exposure in non-human primates.

| **Species** | **Drug/Route/Duration** | **Dose in mg/kg** | **N** | **N with TD** | **Delay of TD induction** | **Other observations** | **Reference** |
| --- | --- | --- | --- | --- | --- | --- | --- |
| Macaca mulatta  (rhesus) | Chlorpromazine / oral /  20 months | 30  (daily) | 17  (both sexes) | 0 | --- | Moderate slowing; acute dystonic reactions in few, responsive to biperiden | [83] |
|  | Haloperidol / oral | Up to approx. 0.5  (daily) | 15 | 0 | --- | Slowing and sedation  No withdrawal TD | [53] |
|  | Chlorpromazine / oral / 3-9 months | 30  (daily) | 10 | 4 | Within 9 months |  |  |
|  | Haloperidol / non-depot i.m. / 6 months | 0.25  (daily) | 6 | 1 | 2 months | Acute dystonic reactions; parkinsonism; persistent oral TD even after drug stopped | [56] |
|  | Chlorpromazine / oral / 113 weeks | 8-40  (daily) | 4 (young) | 4 | ?  (measured at 64 weeks) | Decreased activity; dyskinesias during drug treatment ; persistence documented 12 weeks off-drug | [84] |
|  | Haloperidol / oral / 6 months | 0.2  (daily in two doses) | 4 | 0 | --- | No extrapyramidal reactions | [85] |
|  | Clozapine / oral / 6 months | 5.2  (daily in two doses) | 4 | 0 | --- |  |  |
|  | Remoxipride / oral /  6 months | 3.7  (daily in two doses) | 4 | 0 | --- |  |  |
|  | Eight different antipsychotics / oral / 6 months | Drug-dependent  (daily in two doses) | 16  (2 per drug) | 0 | --- | No extrapyramidal reactions | [86] |
|  | Haloperidol / oral / 6 months | 0.14  (daily) | 5 | 0 | --- | No extrapyramidal reactions | [87] |
|  | Clozapine / oral / 6 months | 5.2  (daily) | 5 | 0 | --- |  |  |
|  | Haloperidol / oral / 6 months | 0.14  (daily) | 5 (3M:2F) | 0 | --- | No extrapyramidal reactions | [88] |
|  | Clozapine / oral / 6 months | 5.2 (daily) | 5 (3M:2F) | 0 | --- |  |  |
| Macaca speciosa (stump-tailed) | Fluphenazine decanoate / i.m. with | 25.0  (every 2 wks) | 8 (5F:3M) | 1  (old male) | upon drug withdrawal | Parkinsonism during drug exposure in 3; 3 deaths; 2 with self-mutilations | [65] |
|  | Haloperidol / i.m. ;  switched to | 1. (daily) |  |  |  |  |  |
|  | Fluphenazine enanthate / i.m.  with | 3.2  (every 2 wks) |  |  |  |  |  |
|  | Haloperidol / oral / 5 yrs | 6.4 (daily) |  |  |  |  |  |
| Macaca fascicularis  (cynomolgus) | Haloperidol / oral / 4‑16 months | 0.5-8  (daily) | 2 | 0 | --- | Sedation & mild parkinsonism | [54] |
|  | Haloperidol / oral / | 5.8-6.8  (daily in 2 doses) | 6M | 0 | --- | Some bradykinesia | [89] |
|  | Olanzapine / oral /  17-27 months | 2.7-3.2 (daily in 2 doses) | 6M | 0 |  |  |  |
| Papio ursinus (baboon) | Dehydration product of haloperidol(HPTP) / i.m. / 43 weeks | 8  (3x/wk) with rest of 8 wks mid-exposure | 4M | 4 | 17 weeks | Orofacial dyskinesia and dystonia, persisting for 9-18 wks after drug withdrawal (until sacrifice) | [58] |
| Saimiri sciureus (squirrel) | Haloperidol / oral / 8‑13 months | 0.5 (daily) | 5 | 0 | --- | Acute dystonic reactions | [90] |
| Cebus  apella  (capuchin) | Haloperidol / oral / 4‑16 months | 0.5  (daily) | 3 | 2 | 3, 12 months | Acute dystonic reactions and parkinsonism in all; TD with oral features, transiently improved by haloperidol, worsened by anticholinergic | [54] |
|  | Haloperidol / oral / 1 yr | 0.5 down to 0.1  (daily) | 2 (1F:1M) | 2 | Within 1 yr | Acute dystonic reactions; gradual decrease in TD intensity after drug withdrawal; TD briefly ameliorated with acute neuroleptic treatment | [55] |
|  | Haloperidol / oral / 8 to 32 months | 0.5-1.0  (daily, with long interruption) | 3 | 2 | 14, 28 months | Acute dystonic reactions | [90] |
|  | Haloperidol / oral / 3-35 months | 0.05-1.0  (daily) | 11 | 4 | 3, 4, 14, 34 months | Acute dystonic reactions in all; 2 with oral TD | [91] |
|  | Fluphenazine enanthate / i.m. / 1 yr | 0.1-3.2  (every 2 wks with interruptions) | 3 middle-aged to old females | 3 | Within 1 yr | Acute dystonic reactions; persistent withdrawal TD, abolished by drug resumption; exacerbation with stress; 1 with oral TD | [92] |
|  | Haloperidol decanoate / i.m./ 3-6 yrs | 1-10 (every 3 wks) | 9  (both sexes) | 3 | 3-6 yrs | Generalized TD (one with oral TD; one with unilateral TD) | [80] |
|  | Fluphenazine decanoate / i.m. / 3-6 yrs | 4-10 (every 3 wks) | 3  (both sexes) | 3 |  |  |  |
|  | Fluphenazine decanoate / i.m. / 48 wks | 0.22 (every 3 wks for 24 wks)  then 0.33 (every 3 wks for 24 wks) consecutively | 19 (15F) | 6 | After drug withdrawal only | All with sedation, bradykinesia; acute dystonia & dyskinesia; 6/19 with withdrawal oral TD, which abated within 1 yr | [64] |
|  | Fluphenazine enanthate / i.m. / 1-3 yrs | 0.25-1.0 (weekly) | 9 (6F) | 4 | 3-35 months  (mean 17) | Acute dystonic reactions in 11; 6/7 with oral TD; 2 deaths | Personal data |
|  | Haloperidol decanoate / i.m. / 1-3 yrs | 0.1-1.2  (weekly) | 9F | 3 |  |  |  |
| Callithrix jacchus (marmoset) | Haloperidol decanoate / i.m. / over 1 yr | 5-15  (monthly for 1 yr ; then interruptions every 3 months) | 13  (both sexes) | 12 | 2.5-14 months (mean 0.6 yr) | TD reduction after acute non-depot haloperidol injections, which triggered acute dystonic reactions in all; TD persistence for at least 5 months after drug withdrawal; 1 death | [62] |
|  | Haloperidol / oral / 2 yrs | 0.25-2.0 (daily, with interruptions) | 4 | 0 | --- | Akinetic / cataleptic syndrome for 4-6 hrs with each dose | [63] |
